# Evaluation of the dose calculation accuracy in intensity‐modulated radiation therapy for mesothelioma, focusing on low doses to the contralateral lung

**DOI:** 10.1120/jacmp.v10i2.2850

**Published:** 2009-04-28

**Authors:** Laurence E Court, David Ching, Deborah Schofield, Maria Czerminska, Aaron M Allen

**Affiliations:** ^1^ Department of Radiation Oncology Dana‐Farber Cancer Institute/Brigham & Women's Hospital Boston MA USA

**Keywords:** mesothelioma, Monte Carlo, IMRT

## Abstract

This study compares Monte Carlo (MC) with conventional treatment planning system (TPS) calculations. The EGS4nrc MC code, BEAMnrc, was commissioned to simulate a Varian 21Ex Linac. The accuracy of the simulations, including points blocked by the jaws, was evaluated by comparing MC with ion chamber and MOSFET measurements. Eight mesothelioma IMRT cases were planned using Eclipse (pencil beam and superposition convolution algorithms). Dose distributions were recalculated using BEAMnrc/DOSxyz, and compared with TPS. MC agreed with experimental results for IMRT fields within 3% (96% of points). For regions blocked by the jaws, average agreement between MC and experiment was better than 5% up to 20 cm from isocenter. The pencil beam algorithm underestimated lung MLD, V20, and V5, compared with MC, by a mean (range) of 16% (11–22%), 9.0% (2.4 – 30.1%), and 11.8% (2 – 30%), respectively. The superposition convolution algorithm gave better agreement of 8.5% (0 – 17%), 4% (0 – 12%) and 0% (–6 – 6%). Mean dose to the targets was better than ±5% in all cases. In conclusion, there is excellent correlation between TPS and MC calculations for the target doses. The pencil beam algorithm and superposition convolution algorithms both underestimate lung dose parameters, but the superposition convolution dose offers improvements in dose calculation accuracy for these patients.

PACS number: 87.55.kh, 87.55.dk, 87.55.Qr

## I. INTRODUCTION

Intensity modulated radiation therapy (IMRT) allows dose distributions unachievable with conventional radiotherapy techniques, and is well suited to treating targets that are geometrically complex, including concave shapes, and situated close to critical targets. Thus, IMRT would appear to be an ideal treatment technique for treating pleural mesothelioma after extrapleural pneumonectomy (EPP), for which the target includes the entire pleural cavity and ipsilateral mediastinal lymph nodes.^(^ ^1^ ^–^ ^5^ ^)^ Importantly, the use of IMRT also allows the avoidance of many important nearby critical structures, including contralateral lung, contralateral kidney, liver, heart and spinal cord. Despite this early promise, IMRT in combination with adjuvant chemotherapy has recently been shown to result in a high rate of treatment‐related fatal pneumonitis.^(3)^ ^,^ ^(6)^ In this experience and others, it was shown that the mean lung dose (MLD) to the contralateral lung was paramount to reducing the pneumonitis risk. Also, it has been suggested, although not proven, that the volume receiving low dose radiation as measured by metrics such as the volume of lung receiving more than 5 Gy (V5) may be the most critical factor in limiting toxicity.^(3)^ ^,^ ^(7)^

The emphasis on low dose to the contralateral lung raises a difficult problem in radiotherapy. Accurate calculation by the treatment planning system (TPS) of IMRT for mesothelioma is a challenge because of three complicating factors. First, there is a difficulty in including the impact of density heterogeneities, particularly lateral electronic disequilibrium. Many groups have investigated the effect of low‐density heterogeneities on dose distributions and calculations, showing that (depending on the anatomical site, correction algorithm, energy, and field size) agreement between experiment (or Monte Carlo simulation) and TPS calculation can be 10% to 20%, or more.^(^ ^8^ ^–^ ^16^ ^)^ Secondly, there are challenges in modeling the multileaf collimators (MLCs). Several groups have shown that inadequate modeling of the IMRT fluences, especially the MLCs, can have a noticeable impact on the accuracy of the calculated doses.^(17)^ ^,^ ^(18)^ Jang et al.^(18)^ attributed TPS underestimates of dose in low dose regions to issues in MLC modeling. Court et al.^(17)^ also found disagreement between Monte Carlo simulations, ion chamber measurements, and TPS calculations, which they attributed to imperfect modeling of the MLCs in the TPS. Finally, and perhaps most importantly in this case, is the difficulty in modeling out‐of‐field doses. In IMRT for mesothelioma, we chose gantry angles and collimator positions which keep as much of the lung out of the field as possible. Much of the dose to the contralateral lung is, therefore, either exit‐dose, or out‐of‐field dose, due to particles scattered from the treatment volume, from the treatment head, and from jaw and MLC transmission.

It is generally accepted that out‐of‐field dose calculations are poor and, although there are various studies measuring or simulating out‐of‐field dose,^(15)^ ^,^ ^(19)^ ^,^ ^(20)^ relatively little attention has been paid to TPS dose calculation accuracy for this region. When calculating doses to the contralateral lung, it is important that we understand the accuracy of out‐of‐field dose calculations.

The purpose of this work was to use Monte Carlo simulations to evaluate the accuracy of TPS calculations for contralateral lung dose, and other critical organs and targets, when using IMRT for mesothelioma. In particular, we investigated the accuracy of contralateral lung dose parameters such as mean lung dose (MLD), the volume of lung receiving 20 Gy or more (V20), and the volume of lung receiving 5 Gy or more (V5).

## II. MATERIALS AND METHODS

### A. IMRT plans

This work used CT images and contours for four patients previously treated with IMRT for mesothelioma following EPP at the Dana‐Farber Cancer Institute / Brigham & Women's Hospital, with IRB approval. The planning techniques are described in detail in the paper by Allen et al.^(5)^ and are summarized here. Two patients were right‐sided treatments and two were left‐sided treatments. The patients had been contoured to include the following structures:

- Clinical target volume 54 (CTV54); this includes all areas of preoperative pleural surfaces, ipsilateral mediastinal lymph nodes, and rectocrural space (as defined by Ahamad et al.^(2)^)
- Clinical target volume 48 (CTV48); this was an additional lower dose CTV used for left‐sided patients, covering regions that were close to the cord or heart to allow sparing of these organs
- Boost gross tumor volume 60 (GTV60); this volume was defined based on areas of PET‐avidity following surgery
- Critical structures: contralateral lung, contralateral kidney, liver, heart, spinal cord

The dose prescription was 54Gy to CTV54, 48Gy to CTV48, and 60Gy to GTV60, all treated simultaneously, with GTV60 receiving 2. 0Gy per daily fraction.

For each patient, two dynamic IMRT plans were created, giving a total of 8 IMRT plans. That is, there were two plan types (A and B), with different lung tolerances and different gantry angles. Type A represented the actual treatment plans described in Allen et al.,^(3)^ whereas the “B” plans represent the restricted field IMRT technique described in the paper by Allen et al.^(5)^ The tolerances used for the normal structures are given in Table 1. Note that for group B, the lung tolerances are somewhat tighter than more traditional metrics. Also note the inclusion of V5 as a dose‐volume constraint. These were research plans, not the plans used to treat the patients.

**Table 1:** Dose‐volume constraints used in the IMRT planning process

*Normal tissue*	*Dose‐volume constraints*	
Contralateral lung	Group A: V20<20%, no V5 constraint, MLD<15Gy	Group B: V20<10%, V5<60%, MLD<9.5Gy
Contralateral kidney	V15<15%	
Liver	V30<30%, Mean liver dose>31Gy	
Heart	V45<30%,V50<20%, Maximum dose 60Gy	
Spinal cord	V45<10%, Maximum dose 50Gy	

The IMRT plans were created using the Eclipse TPS (Varian Medical Systems Inc., Palo Alto, CA). The plans had 7–9 gantry angles, with most beams entering from the ipsilateral side of the patient. Group A had 80°–90° of the contralateral side free from entrance beams. Typical gantry angles for a 9‐field left‐sided treatment were 300, 350, 40, 90, 140, 190, 240 degrees. Group B had 160° of the contralateral side free from entrance beams. Typical gantry angles for a 9‐field left‐sided treatment were 350, 15, 40, 65, 90, 115, 140, 165, and 190 degrees. All final dose distributions were calculated using (a) the modified‐Batho pencil‐beam dose algorithm, and (b) the superposition convolution algorithm (AAA, Anisotropic Analytical Algorithm).

### B. Monte Carlo tools

The EGS4nrc MC code, BEAMnrc,^(21)^ was used to simulate the 6MV X‐ray beam from a 21Ex Linac (Varian Oncology Systems, Palo Alto, CA), and dose distributions were calculated on the patient CT scans using the DOSXYZnrc code. During the initial commissioning of the Monte Carlo code, the parameters of the LINAC model were adapted until open field percentage depth doses matched those measured using a Wellhoffer water tank within 1–2%. After this initial commissioning, the LINAC parameters in the code were kept constant, and the ability of the Monte Carlo code to simulate IMRT treatments and doses outside the field was evaluated. We used cubic pixels with 5 mm sides. ECUT and PCUT values were 0. 7MeV and 0.01MeV, respectively. Monte Carlo simulations used 1 billion particles.

The dynamic MLC component module DYNVLMC^(22)^ was used to fully model the Varian Millennium 120 leaf collimator. This model includes most of the geometric details of the MLC, including leaf tip shape and even the leaf driving screw hole. Published results show that a fully commissioned BEAMnrc with the DYNVLMC module is capable of simulating dynamic IMRT dose distributions that agree with ion chamber within 1%.^(22)^

We previously evaluated the ability of our Monte Carlo code to simulate an IMRT treatment on a Varian 21Ex.^(17)^ These results are summarized here. The code was used to simulate nine split‐field IMRT fields applied en‐face to a $30\times 30\times 16\;{\text{cm}}^{3}$ solid water phantom. The phantom was treated using a Varian 21Ex, and dose at the isocenter plane was measured along a RL direction using 9 calibrated MOSFETs (Thomson‐Nielson; Ottawa, ON, Canada) spaced every 2.5 cm. The Monte Carlo code was further tested by simulating the dose distributions for an IMRT plan previously calculated for the anthropomorphic head and neck phantom from the Radiological Physics Center (RPC) (University of Texas M. D. Anderson Cancer Center) as part of the credentialing process needed to enter patients into certain protocols that allow the use of IMRT. This phantom includes TLDs that staff at RPC analyses to compare delivered and calculated doses. These tests evaluate the use of Monte Carlo to model doses in the inner beam region when using moving MLCs.

For the current study, it was necessary to supplement this data and evaluate the accuracy of Monte Carlo dose simulations outside the collimators. This is important because, for much of the treatment, the contralateral lung is outside the collimators. First the inner beam, penumbral region, and the outer beam up to 5 cm outside the jaw edges were evaluated for square symmetric fields with side dimension 4 cm, 6 cm, and 10 cm. Dose profiles at depths of 1.6 cm, 5 cm, 10 cm, 20 cm, and 30 cm calculated with Monte Carlo were compared with profiles measured using an ion chamber (CC13 [0.13 cm^3^], Scanditronix Welhoffer, Schwarzenbruck, Germany) in a Wellhoffer water tank.

The accuracy of Monte Carlo dose calculations for the outer region more distant from the collimators, starting 5 cm outside the collimators up to 30 cm from the isocenter, was then evaluated. A solid water phantom with dimensions 60 cm×30 cm×14 cm (RL×SI×AP) was used. Isocenter was set at a point 7 cm deep, and 15 cm from three of the sides. An ion chamber (A12 [0.65 cm^3^], Standard Imaging, Middleton, WI) was then used to measure dose at the isocenter plane at 10 cm, 13 cm, 16 cm, 20 cm, 25 cm and 30 cm from isocenter. Monte Carlo calculations were performed for the same geometry. This was repeated for the following field sizes: 10cm × 10cm defined by the jaws, 10cm×10cm defined by both jaws and MLCs, and 10×10 cm defined with MLCs with jaws set to 20×20 cm. Monte Carlo results were fitted to a local exponential formula, and the fitted data compared with the experimental data. These combinations represent the different combinations that might be found in real clinical cases where distant doses may include contributions from transmission through jaws, MLCs, and both jaws and MLCs.

To illustrate the dose calculation issues, in all cases the above dose distributions were also calculated using the TPS (pencil‐beam and superposition convolution algorithms), and compared with Monte Carlo and experimental data.

Note: these tests did not include any significant density heterogeneities and do not, therefore, test the ability of the Monte Carlo code to evaluate the impact of tissue heterogeneities. Heath et al.^(22)^ have shown that simulations using this code for heterogeneous phantoms, treated with simple jaw‐defined fields, agree with TLD and parallel‐plate ion chamber measurements within 2% and 1% on average, respectively. Other researchers have reported similar agreement between this Monte Carlo code and measurements in heterogeneous phantoms.^(9)^ ^,^ ^(23)^ ^,^ ^(24)^ Monte Carlo results from this work are dose to medium. The difference between dose to medium and dose to water for soft tissue, including lung, as been shown to be 1% or less.^(25)^

### C. IMRT plan comparison

The Monte Carlo code was used to simulate the dose distributions for the 8 IMRT plans described above. The plans were then compared with the dose distributions calculated using the TPS. Statistical uncertainty of the Monte Carlo dose was approximately 0.5% of the local dose. Comparisons were made using profiles, the DVH metrics listed in Table 1, target coverage (percentage volume receiving 95% of the prescribed dose), and the mean dose for each target. In all comparisons, volume comparisons (change in coverage or V5, etc.) are expressed as a percentage of the total structure volume. Dose comparisons (e.g. MLD) are expressed as the percentage change, referenced to the Monte Carlo calculated dose.

## III. RESULTS

### A. Commissioning of the Monte Carlo software

Comparison of the doses calculated on the solid water phantom using MC, compared with doses measured using MOSFETs (total 81 points) showed mean difference, standard deviation, and range of –0.03%, 1.26%, and –5.6% to 2.5%, respectively, when expressed as a percentage of the 200cGy prescription dose. Only 3 points (4% of points) showed agreement worse than ±3%. Agreement between TLD results and Monte Carlo results for the head phantom was within 2.0% (these initial commissioning results were previously reported in Court et al.^(17)^).

**Figure 1:**
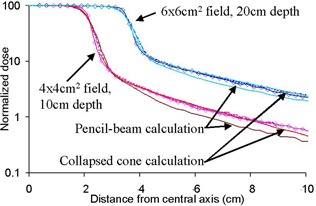
Comparison of Monte Carlo simulations (diamonds), experimental data (dotted lines) and TPS calculations (solid lines) for the central field and outer region. Two comparisons are shown: a 4×4 cm field at 10 cm depth and a 6×6 cm field at 20 cm depth. Doses are normalized at central axis. It can be seen that Monte Carlo and experimental data agree, and that the TPS underestimates dose outside the field.

Sample comparisons of Monte Carlo simulations, experimental data, and TPS calculations up to 5 cm from the collimator edge are shown in Fig. 1. Agreement between Monte Carlo simulations and experimental measurements for points in the penumbra agreed within 2 mm distance‐to‐agreement for the high dose regions. For lower dose regions, average agreement was ±3%, with Monte Carlo tending to give slightly lower doses than measured.

The average agreement between Monte Carlo and experimental data for the region distant from the collimators, up to 20cm from isocenter, expressed as a percentage of the local experimental measurement was 5±1%, 2±5%, and 3±2% when the field size was 10 cm×10 cm defined by the jaws, 10×10 cm defined by both jaws and MLCs, and 10×10 cm defined with MLCs with jaws set to 20×20 cm, respectively. For this region, Monte Carlo tended to give slightly lower doses than those measured. For points further than 20 cm from isocenter, the differences were generally larger, depending on the radiation conditions, at 30%, 31% and 1%, for the same fields, respectively. It can be seen in Fig. 1 that the pencil‐beam algorithm underestimates the dose for all points outside the field, with the magnitude of the difference depending on the radiation conditions and distance from the field edge. The superposition convolution algorithm models dose outside the field much more accurately. It can also be seen that the Monte Carlo results are consistently closer to the measured data than the TPS calculated doses.

### B. Comparison of TPS and Monte Carlo dose calculations

Figure 2 shows two typical axial slices (TPS dose calculations) indicating two lateral lines through which dose profiles are compared. There is good agreement in the high dose (target) regions, but the TPS underestimates the doses in lower dose regions, relative to Monte Carlo, particularly outside the fields.

**Figure 2(a) and (b):**
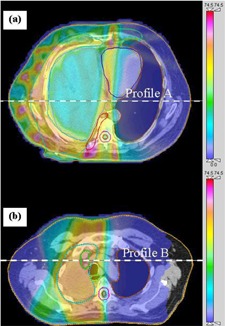
Two axial slices with dose distribution calculated using the TPS. The locations of the two profiles of are shown.

Figure 3 shows a typical lung DVH for a left‐sided treatment as calculated from Monte Carlo simulations, Eclipse pencil‐beam and Eclipse superposition convolution algorithm. In this case, the AAA algorithm and Monte Carlo gave good agreement, but the pencil‐beam gave lower lung doses. More details are given in Table 2, which compares the dose distribution parameters for the critical organs. In general, the superposition convolution algorithm showed better agreement with the Monte Carlo simulations than did the pencil‐beam algorithm. In particular, the pencil‐beam algorithm underestimated all the important lung parameters. A Wilcoxon's matched pairs signed rank test showed that the underestimation of dose found by the TPS compared with the Monte Carlo simulation was significant at the 5% level for MLD, lung V5, lung V20, and mean liver dose for the pencil‐beam algorithm, and for MLD and lung V20 for the superposition algorithm.

**Figure 2(c):**
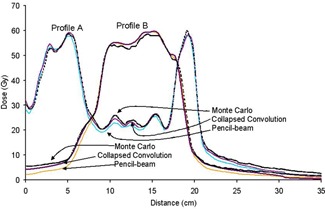
Lateral profiles along the lines shown in slice A and B (and (b)) showing TPS and MC results. Dotted lines are MC, solid lines are TPS. In all cases, TPS dose calculations are lower outside the field compared with Monte Carlo calculations.

**Table 2:** Comparison of dose distribution parameters calculated using the pencil‐beam and superposition convolution algorithms with Monte Carlo results. Volume comparisons (change in coverage or V5, etc.) are expressed as a percentage of the total structure volume. Dose comparisons (e.g. MLD) are expressed as the percentage change, referenced to the Monte Carlo calculated dose. A positive value means the Monte Carlo simulation showed a higher dose or volume than the TPS calculation.

*Tissue*	*Mean difference between TPS calculation and MC simulation (range)*	
	*All are in percent (%)*	
	*Pencil‐beam algorithm*	*Superposition convolution algorithm*
Lung mean dose	16.4 (11.5 – 22.5)	8.5 (0.4 – 17.2)
Lung V5	11.8 (2.4 – 30.1)	‐0.2 (–6.4 – 5.6)
Lung V20	9.0 (6.5 – 16.4)	4.1 (0.0 – 11.5)
Liver mean dose	3.7 (0.9 – 8.0)	2.1 (–2.3 – 6.9)
Liver V30	3.0 (–0.2 – 10.8)	0.0 (–1.8 – 3.7)
Heart V45	2.8 (–6.0 – 11.5)	–1.8 (–6.0 – 1.7)

Differences in the dose calculations for the targets were relatively small. Mean differences in mean dose calculated for the GTV and CTV were less than 1% for both algorithms (standard deviation: 2.5%). Mean change in coverage (volume receiving 95% of prescription) was 1±2.5% and 0.5±2.4% for the pencil‐beam and superposition convolution algorithms, respectively. Coverage was reduced by more than 5% (volume) for one of the targets in 3 plans, and increased by more than 5% for 2 plans, but these changes were caused by the steep gradients of DVHs in this region for these plans.

**Figure 3:**
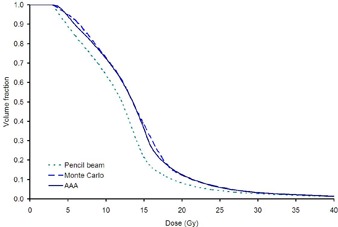
A typical lung DVH for a left‐sided treatment, as calculated using the three methods.

## IV. DISCUSSION

Many TPS are designed to and can calculate delivered dose within 5% accuracy for most planning situations, including the presence of density heterogeneities. It is very important, however, to understand these calculation models and TPS may not be as robust when diverging from accepted treatment techniques (e.g. IMRT with multiple fields vs. APPA treatments), or when looking at new evaluation parameters (e.g. lung V5 vs. the more common V20), or – most notably–when evaluating doses to avoidance structures far “out of field”. It is also important to realize that current IMRT QA techniques will not necessarily catch these calculation errors. For example, IMRT QA using ion chamber typically measures doses delivered to the primary targets; film dosimetry may not be reliable for very low dose regions. Understanding this quandary necessitates an alternative, more robust, system to ensure that these complex plans when created are being reliably delivered.

This study compared the pencil‐beam dose calculation algorithm (modified‐Batho heterogeneity correction) and superposition convolution algorithm (as implemented in Eclipse) with Monte Carlo simulations. Both were adequate for calculating doses to the targets, but both underestimated doses delivered to critical organs (i.e. the contralateral lung) which, depending on the field, lay outside the open jaws or were in the exit‐dose region.

These differences can be attributed to how well (if at all) the algorithms treat scatter from the jaws and MLCs, and scatter from the target volume to distant points. Agreement between Monte Carlo simulations and the superposition convolution algorithm was better than between the Monte Carlo simulations and pencil‐beam algorithm. This important difference must be considered as clinics which previously used the pencil‐beam algorithm move to implement the superposition convolution algorithm.

The broader implications of these findings are quite significant. With clinical implementation of IMRT for thoracic tumors becoming widespread in clinics across the country, the accuracy of TPS IMRT calculations is critical, as is an understanding of the differences between different algorithms. While some have reported decreased thoracic complications with thoracic IMRT,^(26)^ ^,^ ^(27)^ others have reported increased pneumonitis with IMRT for NSCLC.^(28)^ ^,^ ^(29)^ In these reports, researchers raised concerns about the importance of low dose radiotherapy in the lungs and the accuracy of TPS calculations.^(28)^ ^,^ ^(29)^ It is important, therefore, to understand the limitations of our dose calculations.

## V. CONCLUSIONS

For the eight cases evaluated in this study, both superposition convolution and pencil‐beam algorithms underestimate important lung dose parameters in mesothelioma IMRT when compared with Monte Carlo calculations. The pencil‐beam algorithm gives particularly large differences, with mean lung dose underestimated by up to 22%, and the volume receiving 5Gy underestimated by up to 30%. These worst‐case errors are reduced to 17% and 5.6%, respectively, when the superposition convolution algorithm is used. Both algorithms accurately calculate dose to the targets.

## CONFLICTS OF INTEREST NOTIFICATION

The authors report no actual or potential conflicts of interest.
